# Endoscopic ultrasound-guided fine-needle aspiration in diagnosing primary medistinal large B-cell lymphoma: a case report

**DOI:** 10.3389/fonc.2025.1404211

**Published:** 2025-03-25

**Authors:** Jingyan Tang, Yuchen Guan, Jianfeng Zhang, Chengqi Guan

**Affiliations:** Department of Gastroenterology, Affiliated Hospital of Nantong University, Nantong, China

**Keywords:** endoscopic ultrasound-guided fine-needle aspiration (EUS-FNA), primary mediastinal large B cell lymphoma (PMBCL), dysphagia, diagnosis, case report

## Abstract

**Background:**

Mediastinal tumors present diagnostic challenges due to their unique location. This case report presents a patient diagnosed with primary mediastinal large B-cell lymphoma (PMBCL) using endoscopic ultrasound-guided fine needle aspiration (EUS-FNA), demonstrating the utility of this minimally invasive technique in detecting and confirming PMBCL.

**Case description:**

A 34-year-old previously healthy woman came to our hospital complaining of dysphagia for 3 months. The gastroscopy showed a huge submucosal bulge in the middle of the esophagus, and a contrast-enhanced computed tomography scan of the chest revealed a left main bronchus nodule measuring 15 mm, mediastinal lymph node enlargement, and fusion with necrosis. Subsequently, we obtained the tissue from the mediastinal mass through EUS-FNA and the tissue from the left main bronchus nodule through transbronchoscope biopsy. According to the pathologic findings, we made a clear diagnosis: primary mediastinal large B-cell lymphoma.

**Conclusion:**

As a minimally invasive technique, EUS-FNA is highly safe, repeatable, and accurate for lymphoma diagnosis. Although there are some limitations, it can play an important role in diagnosing mediastinal tumors.

## Introduction

Primary mediastinal large B-cell lymphoma (PMBCL) is a rare, aggressive subtype of B-cell lymphoma, comprising approximately 2%–3% of all non-Hodgkin’s lymphomas (NHLs). Characterized by rapidly enlarging anterior mediastinal masses, PMBCL often presents with symptoms like dysphagia or dyspnea due to compression of surrounding structures. The clinical manifestations are rapidly increasing anterior mediastinal masses and longitudinal compression and infiltration of the septal mass on the surrounding organs (1). So, there are some patients with dysphagia or dyspnea as the first symptom. Due to its unique clinical, histological, and molecular characteristics, PMBCL has been listed as a separate type in lymphoma classification by the World Health Organization since 2016 (2). How to diagnose it is very useful for those patients with atypical symptoms. Endoscopic ultrasound-guided fine needle aspiration (EUS-FNA) can effectively alleviate this difficult situation.

## Case description

A 34-year-old previously healthy woman came to our hospital complaining of dysphagia for 3 months. She denied having tarry stool, weight loss, or chest pain. She also denied a family history of gastrointestinal carcinoma or a history of previous surgery. Upon presentation, no positive findings were found on physical examination.

**Figure 1:**
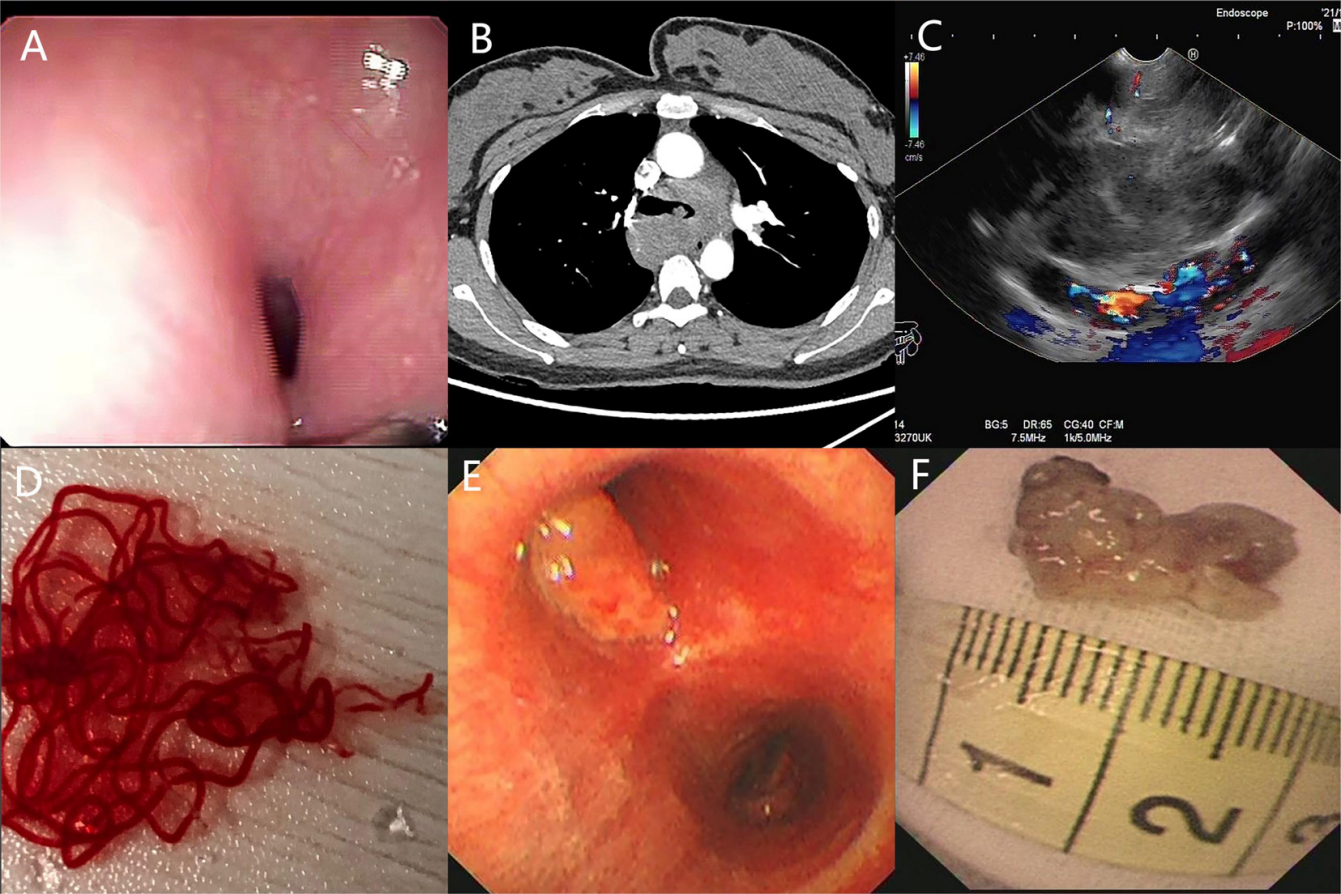
**(A)** Gastroscopy shows a huge submucosal bulge in the middle of the esophagus. **(B)** Contrast-enhanced computed tomography scan of the chest shows a left main bronchus nodule, mediastinal lymph node enlargement, and fusion with necrosis. **(C, D)** Endoscopic ultrasound reveals a 30 × 25-mm hypoechoic lesion arising from the mediastinal mass, and EUS-FNA was performed to obtain tissue from the mass. **(E, F)** Transbronchoscope biopsy was performed to obtain tissue from the left main bronchus nodule.

**Figure 2:**
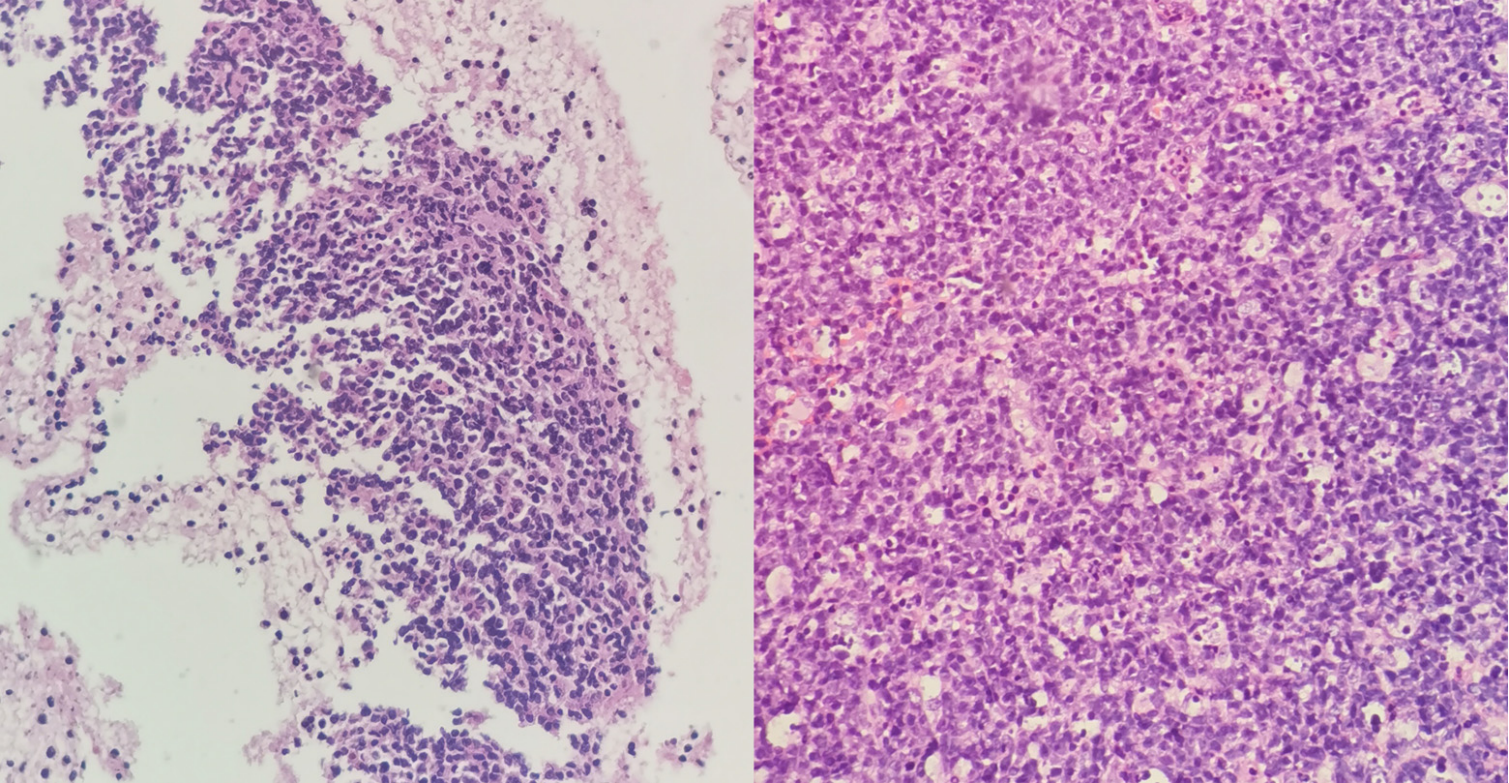
(Left) Hematoxylin and eosin (H&E) shows medium to large tumor cells with transparent cytoplasm of the mediastinal masses. (Right) H&E shows medium to large tumor cells with transparent cytoplasm of the left bronchus nodule.

The patient underwent a series of diagnostic assessments. Initial gastroscopy revealed a significant submucosal bulge in the middle segment of the esophagus (**Figure 1A**). Then, a contrast-enhanced computed tomography scan of the chest showed a left main bronchus nodule measuring 15 mm, mediastinal lymph node enlargement, and fusion with necrosis (**Figure 1B**). Subsequently, we obtained the tissue from the mediastinal mass through EUS-FNA (**Figures 1C, D**) and the tissue from the left main bronchus nodule through transbronchoscope biopsy (**Figures 1E, F**). Both of the samples’ pathological results revealed a large number of tumor cells proliferating diffusely. The tumor cells were medium to large, and the cytoplasm was rich and transparent. The nucleus was round or oval, and the nuclear chromatin consisted of coarse particles or vacuoles. Tumor cells were often divided into nests by proliferating fibrous cords (**Figure 2**). Based on clinical evidence, the patient was considered to have a lymphoma. Due to the complexity of lymphoma classification, different types of lymphoma have different treatment options, and it is very important for patients to clarify the pathological type.

**Figure 3:**
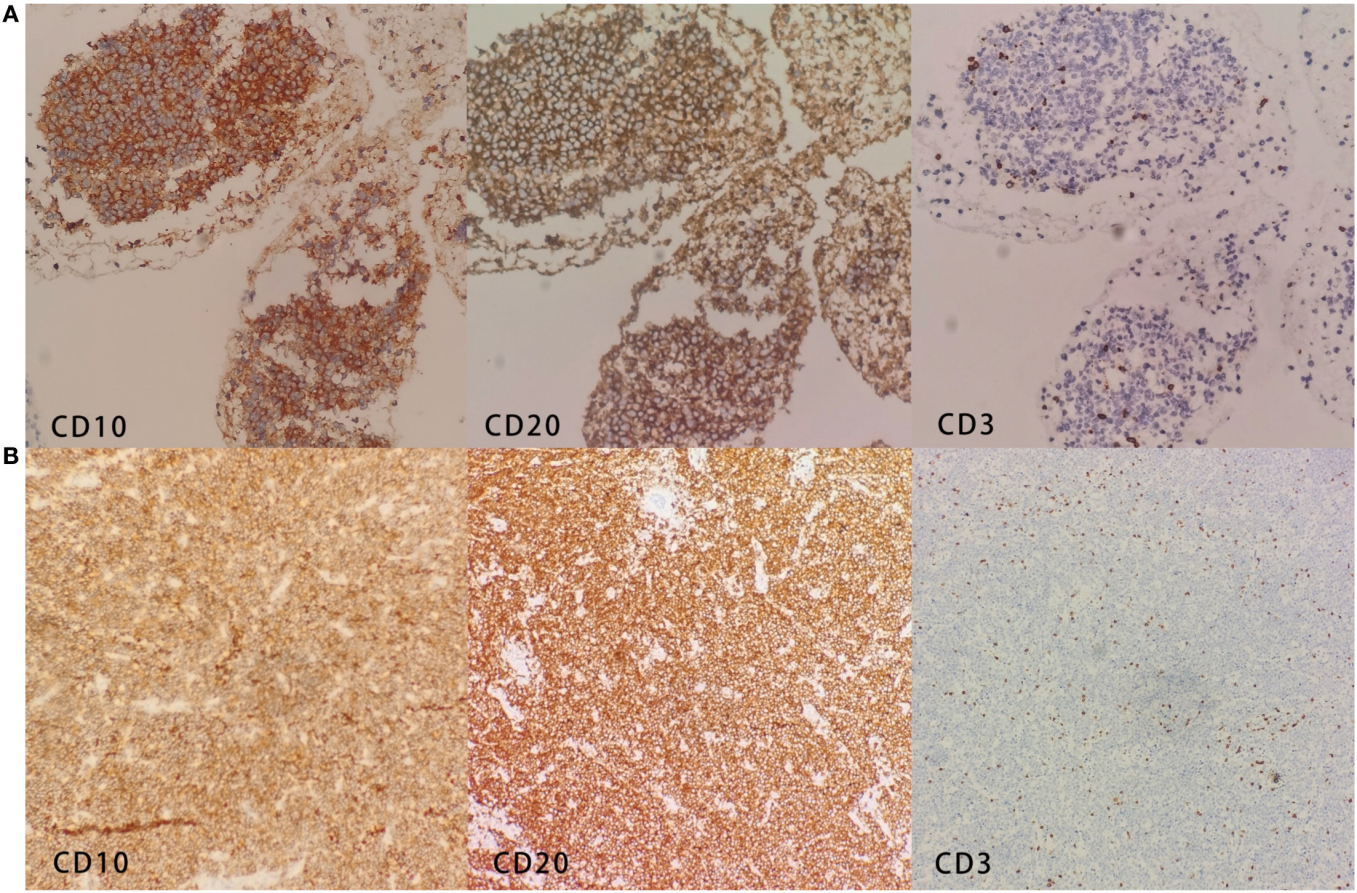
**(A)** Immunohistochemistry of the mediastinal mass shows CD10+, CD20+, and CD3−. **(B)** Immunohistochemistry of the left bronchus nodule shows CD10+, CD20+, and CD3−.

Immunohistochemistry of the mediastinal mass showed that the tumor cells were diffusely and strongly positive for CD10, CD20, CD23, LCA, and Ki67 (80%) and negative for CD3, CK18, CKpan, TTF-1, Syn, CgA, CD5, P63, and Mum-1 among others (**Figure 3A**). Molecular analysis detected a strongly positive SHOX2 gene and a negative RASSF1A gene. Immunohistochemistry of the left bronchus nodule showed that the tumor cells were diffusely and strongly positive for CD10, CD19, CD20, CD22, CD23, Ki67 (90%), and Bcl-6 and negative for CD3, CD5, CD30, EBER, and Mum-1 (**Figure 3B**).

MAL, CD200, and CD23 have been considered as key immunophenotypic markers for the identification of DLBCL and PMBCL (3). The expression of MAL and CD23 in our patient was positive, and PET-CT indicated that there was no distant metastasis, so we can differentiate it from DLBCL. CD20, CD23, CD30, and CD79a were helpful to identify classical Hodgkin’s lymphoma and PMBCL (1, 4). Our patient’s immunohistochemistry showed positive CD20 and CD23 and negative CD30 and Mum-1, so it can be distinguished from classical Hodgkin’s lymphoma. Finally, based on all results of clinical symptoms, pathology tests, and other related examinations, the patient was diagnosed with primary mediastinal large B-cell lymphoma.

**Figure 4:**
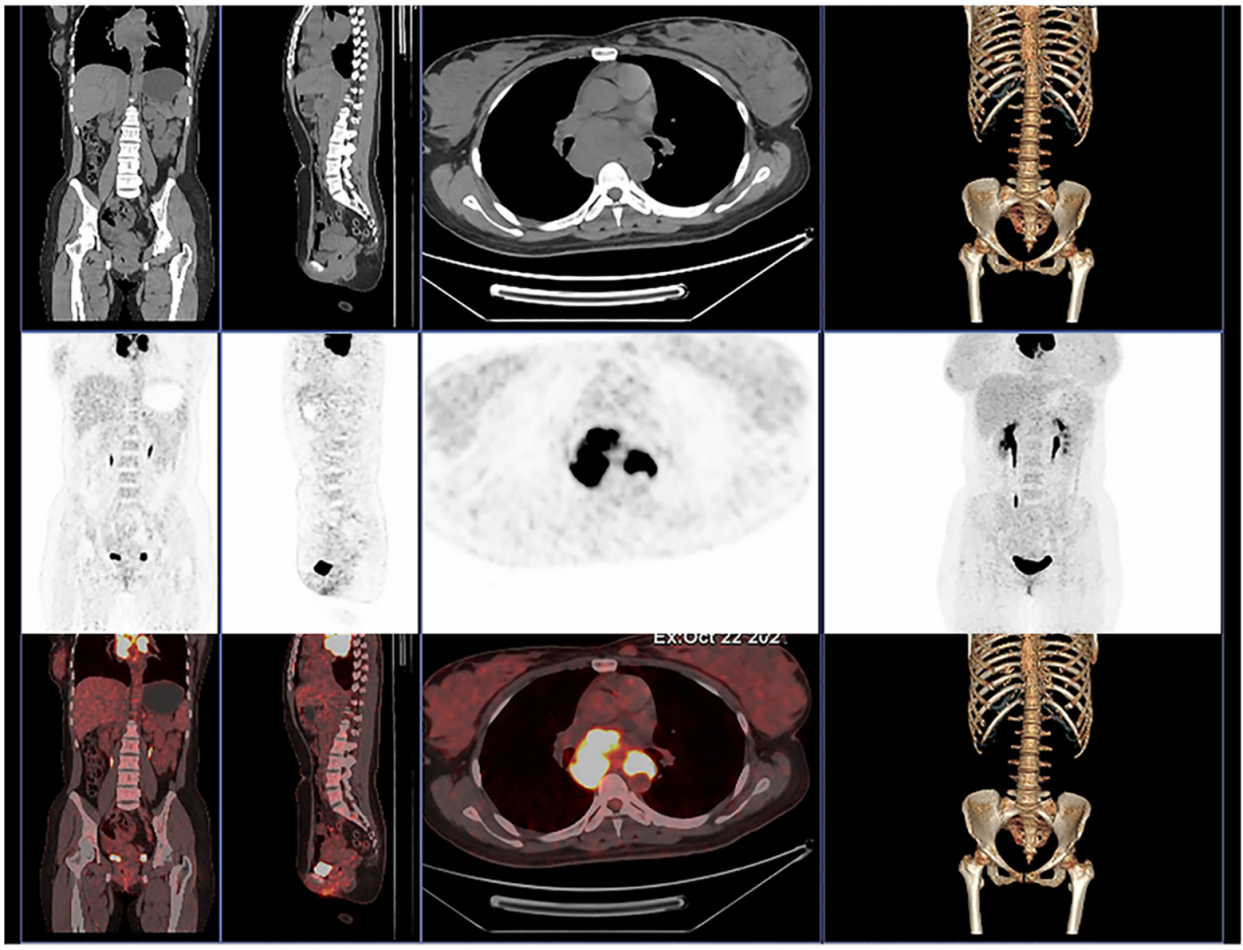
The ^18^F-FDG PET/CT of our patient shows multiple enlarged confluent lymph nodes in the mediastinum with significantly increased glucose metabolism.

Moreover, in order to further evaluate the whole-body condition and choose the treatment, we did 18F-fluorodeoxyglucose positron emission tomography/computed tomography (^18^F-FDG PET/CT) examination on day 12 (**Figure 4**). The results indicated multiple enlarged confluent lymph nodes in the mediastinum with significantly increased glucose metabolism (5).

Then, the patient was transferred to the hematology department for six cycles of R-DA-EPOCH targeted chemotherapy. After the treatment, the clinical symptoms of the patient were relieved and disease progression slowed down. So far, our patient is still in regular follow-up.

## Discussion

The patient presented with dysphagia, prompting an initial evaluation for primary esophageal diseases, including esophageal carcinoma. Endoscopic examination identified abnormal findings, which led to further investigation with EUS-FNA for definitive diagnosis. In order to further explore the etiology, we decided to obtain the pathological type of the unknown mass shown by the esophagoscope, so we used the EUS-FNA technique and successfully obtained the mass sample. Luckily, we confirmed the diagnosis of PMBCL for the patient based on pathological results and other examinations.

PMBCL is thought to originate from thymic B cells, accounting for approximately 10% among all large B-cell lymphomas. It mainly expresses B-cell surface molecules, such as CD10, CD19, CD20, CD22, and CD231. Moreover, it mainly occurs in young adults, with a median age of 35 years, and is more common in women than men (1). Patients may have compression symptoms caused by previous mediastinal masses or superior vena cava syndrome, which is caused by a thrombosis. The common symptoms include cough, dyspnea, hoarseness, dysphagia, and B symptoms (fever, night sweats, and weight loss) (6).

The previous pathological diagnosis of mediastinal masses used to rely on mediastinal biopsy, transbronchial needle aspiration, and CT-guided percutaneous puncture and magnetic resonance imaging. All of these methods can cause severe physical trauma, even respiratory failure, cardiac arrest (7). EUS-FNA can be used for the pathological examination of mediastinal, retroperitoneal, and gastrointestinal lymph nodes, which may be too small or unable to enter using detection techniques (8). Compared with mediastinal biopsy, it is considered to be a relatively safe, fast, and minimally invasive technique with a lower rate of complications and can provide important information for further management of patients (9). In radiology publications, the accuracy and sensitivity of FNA cytology for lymphoma range from 68% to 94% and from 66% to 90%, respectively (10). A relative study showed that the sensitivity, specificity, and accuracy of EUS-FNA combined with flow cytometry for diagnosing lymphoma were 72.7%, 100%, and 89.7%, respectively (11). These lines of evidence show that the potential value of EUS-FNA in diagnosing mediastinal masses is immeasurable in some specific states. However, due to the sample tissues, there is a possibility of false negatives. In a study published by Ribeiro et al., only 29.2% of EUS-FNA results were consistent with standard excisional biopsy (12). In addition, experienced endoscopists are also essential.

## Conclusion

PMBCL is increasingly recognized in clinical practice. For patients with atypical presentations involving other systems, early consideration of PMBCL as a differential diagnosis can prevent delays in diagnosis. EUS-FNA has proven to be a valuable, minimally invasive tool for the diagnosis of anterior mediastinal masses, offering critical insights that guide management.

## Data Availability

The original contributions presented in the study are included in the article/supplementary material. Further inquiries can be directed to the corresponding author.
